# Thermoluminescence Enhancement of LiMgPO_4_ Crystal Host by Tb^3+^ and Tm^3+^ Trivalent Rare-Earth Ions Co-doping

**DOI:** 10.3390/ma12182861

**Published:** 2019-09-05

**Authors:** Wojciech Gieszczyk, Barbara Marczewska, Mariusz Kłosowski, Anna Mrozik, Paweł Bilski, Anna Sas-Bieniarz, Paweł Goj, Paweł Stoch

**Affiliations:** 1 Institute of Nuclear Physics Polish Academy of Sciences, Radzikowskiego 152, PL31342 Krakow, Poland (B.M.) (M.K.) (A.M.) (P.B.) (A.S.-B.); 2 AGH University of Science and Technology, Mickiewicza 30, PL30059 Krakow, Poland (P.G.) (P.S.)

**Keywords:** LiMgPO_4_, LMP, lithium magnesium phosphate, trivalent rare-earth ions, terbium, thulium, luminescence properties, luminescent enhancement, thermoluminescence

## Abstract

We investigated the influence of terbium and thulium trivalent rare-earth (RE) ions co-doping on the luminescent properties enhancement of LiMgPO_4_ (LMP) crystal host. The studied crystals were grown from the melt by micro-pulling-down (MPD) technique. Luminescent properties of the obtained crystals were investigated by thermoluminescence (TL) method. The most favorable properties and the highest luminescence enhancement were measured for Tb and Tm double doped crystals. A similar luminescence level can be also obtained for Tm, B co-doped samples. In this case, however, the low-temperature TL components have a significant contribution. The measured luminescent spectra showed a typical emission of Tb^3+^ and Tm^3+^ ions of an opposite trapping nature, namely the holes and electron-trapping sites, respectively. The most prominent transitions of ^5^D_4_ → ^7^F_3_ (550 nm for Tb^3+^) and ^1^D_2_ → ^3^F_4_ (450 nm for Tm^3+^) were observed. It was also found that Tb^3+^ and Tm^3+^ emissions show temperature dependence in the case of double doped LMP crystal sample, which was not visible in the case of the samples doped with a single RE dopant. At a low temperature range (up to around 290 °C) Tm^3+^ emission was dominant. At higher temperatures, the electrons occupying Tm^3+^ sites started to be released giving rise to emissions from Tb-related recombination centers, and emissions from Tm^3+^ centers simultaneously decreased. At the highest temperatures, emission took place from Tb^3+^ recombination centers, but only from deeper ^5^D_4_ level-related traps which had not been emptied at a lower temperature range.

## 1. Introduction

**Figure 1:**
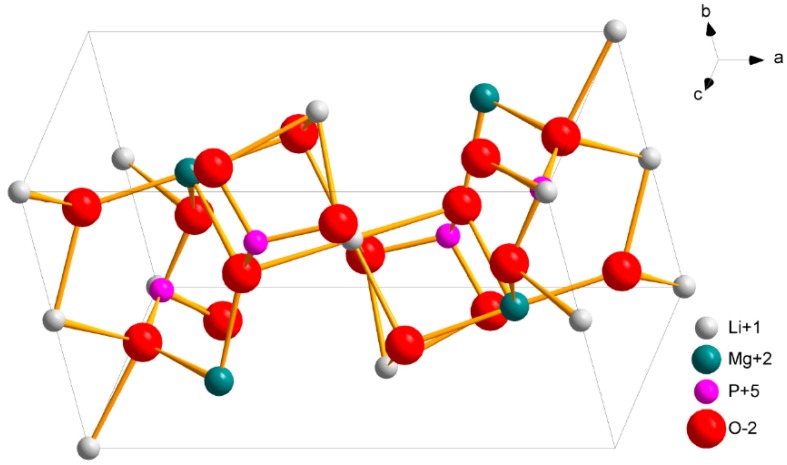
3D crystal structure of LiMgPO_4_ prepared by Diamond 4.1 software using the data from Crystallographic Open Database (COD 1530053). View towards the <h,k,l> = <1,1,1> plane.

LiMgPO_4_ crystallizes in orthorhombic systems where tetrahedral PO_4_, octahedral LiO_6_, and MgO_6_ groups constitute the structure forming a 3D network. This compound belongs to a large family of orthophosphates of general ABPO_4_ formula. Its structure depends on the relative size of the ions, namely, in case of LiMgPO_4_, where the sizes of monovalent A (Li^+^) and divalent B (Mg^2+^) ions are relatively small, the final compound adopts the olivine structure. Li^+^ ions take the positions at the symmetry centers and Mg^2+^ ions occupy the positions on the mirror planes. The structure contains tetrahedral PO_4_ and octahedral MgO_6_ and LiO_6_ groups which share edges and corners. A 3D crystal structure of LiMgPO_4_ obtained with Diamond 4.1. software is shown in Figure 1. More detailed structure specification of the studied compound can be found in [1]. 

The group of 15 elements separated from the sixth period of periodic system is commonly called the rare-earths (RE). Two types of electronic configurations are typical for this group of elements, namely [Xe]4f^n^6s^2^ and [Xe]4f^n-1^5d^1^6s^2^ where [Xe] represents the electronic configuration of the noble gas xenon and *n* denotes the number of electrons occupying the f subshell (from n = 0 for La to n = 14 for Lu). The most common oxidation state of lanthanide ions is 3+, as this state is usually energetically stable. Therefore, the electronic configuration of all the trivalent lanthanide ions is [Xe]4f^n^, which means that the two 6s electrons, as well as the possible 5d electron, have been lost [2]. A characteristic property of the trivalent rare earth ions is that their electronic transitions usually occur within the 4f shell (4f–4f transitions, with an exception of Ce^3+^ where 5d–4f transitions take place). It is important to note that 4f shell is shielded from the host lattice by the optically passive outer electronic shells. This feature reduces the influence of the host lattice on the wavelengths, bandwidths and cross sections of the relevant optical transitions.

The rare-earths doped materials play an important role in the modern optical technology. Their partially filled f-shells, shielded by the outer filled s- and p-shells, allow spectrally narrow electronic transitions at wavelengths ranging from the far infrared to the vacuum ultraviolet. The rare-earth metals have already found a broad range of applications, mainly in modern technologies, e.g., for car catalyst production (La, Ce, Pr, Nd), solid magnets (Pr, Nd, Tb, Dy), optical filters (La, Ce, Pr, Nd), and phosphors (Y, La, Ce, Eu, Gd, Tb, Tm). This last case is nowadays extensively studied regarding the possibility of production of white light emitting diodes (WLED) [3]. The photon upconversion phenomenon, understood as an anti-Stokes photoluminescence process where the energies of two or more sequentially absorbed photons combine to produce a higher energy photon, was also demonstrated by several lanthanide ions, such as Dy^3+^, Ho^3+^, Er^3+^, and Tm^3+^.

Recently, the rare-earths doped luminescent materials are of increasing interest, because of their potential application as the energy storage phosphors for dosimetric applications, as well as the scintillating materials, for which the stage of trapping of free charge carriers is not usually concerned. A lot of attention is now payed for lithium magnesium phosphate (LiMgPO_4_, LMP) compound, as it shows a high radio-sensitivity and a broad linear dose-response range [4]. Since 2010, almost 30 research papers on luminescent properties of differently doped LMP, as a good candidate for application in ionizing radiation dosimetry, have been published [4,5,6,7,8,9,10,11,12,13,14,15,16,17,18,19,20,21,22,23,24,25,26,27,28,29]. Among them, the Tb and B co-doped LMP is most extensively studied [6,7,8,9,11,12,16,17,19,24,26,27,28], however, the other rare-earths dopants, like e.g., Eu [5,13,15,29], Sm [10,15], Tm [23,29], Er [29], and Y [29], were also considered and investigated. In most cases, the samples in form of the powders or cold-pressed and sintered pellets (mixed with polytetrafluoroethylene, PTFE) have been studied, but also the LMP crystals were successfully grown from the melt by micro-pulling-down method [4,18,21,22,29]. The LMP samples in crystal form show considerable advantages over the powders and pellets [22]. Namely, the low-temperature luminescent signal (<150 °C), which dominates the glow-curves measured for powder samples, is not observed (or is strongly reduced) in crystal samples (further proved in this work). This low-temperature luminescence originates from the electrons captured by the shallow electron traps and may have a significant impact to a strong fading observed for this material [26]. The absence of this low-temperature component suggests that high-temperature crystallization (close to the melting point – 1025 °C) dramatically changes the energetic distribution of TL-related structure defects in this material and may lead to the improvement of its dosimetric properties.

**Figure 2:**
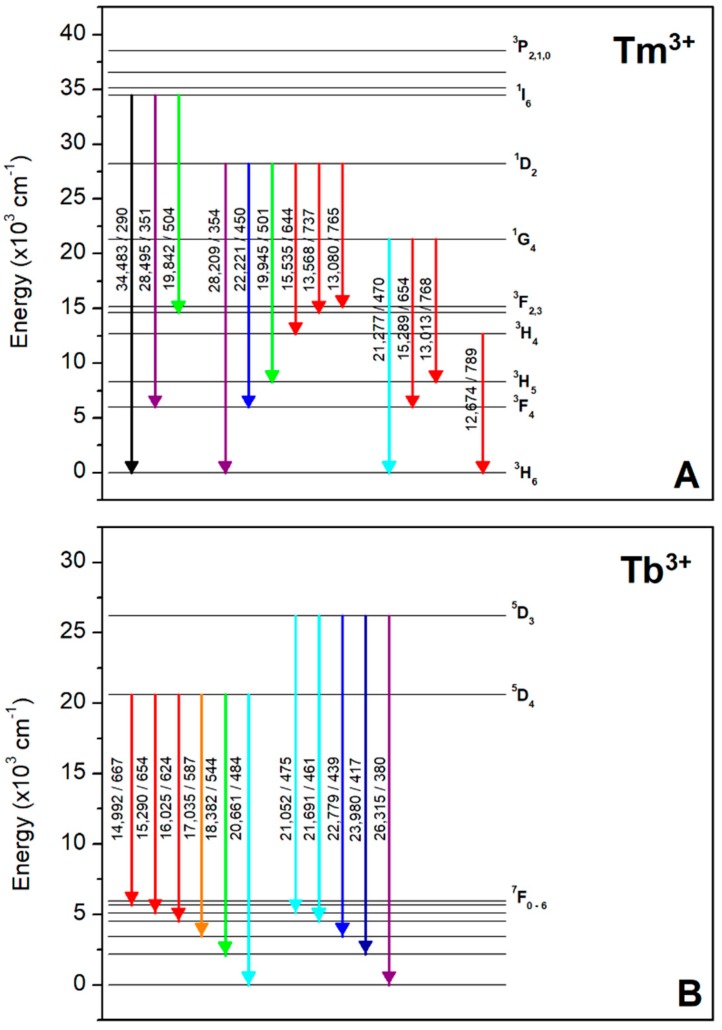
The f–f electronic transitions for trivalent Tm (panel **A**) and Tb (panel **B**) rare-earth ions. The numbers next to arrows denote the energy (cm^−1^) and wavelength (nm) of respective transitions.

As previous investigations [29] showed that the thulium doped LMP crystals possess the highest radio-sensitivity (almost 3 times higher as compared to LiF:Mg,Ti—the gold standard of TL radiation dosimetry), this work is focused on comparative studies of luminescent properties of rare-earth ions co-doped LMP crystals obtained by micro-pulling-down method. The influence of Tb^3+^, Tm^3+^ trivalent rare-earth ions co-doping on luminescence enhancement of LiMgPO_4_ crystal host has been investigated. The measurements were performed by thermoluminescence (TL) method. In most cases, the rare-earth ions substitute the other ions of similar size and the same charge state in the host lattice. Tb^3+^ and Tm^3+^ may potentially replace both the Li^+^ and Mg^2+^ in LiMgPO_4_ lattice, however, significant differences between ionic radii, which are 0.58, 0.72, 0.88, and 0.93 Å for Li^+^, Mg^2+^, Tm^3+^, and Tb^3+^, respectively, may cause a significant lattice distortion or move the doping ions to the interstitial positions. Also, the charge compensation process, commonly manifested in the form of creation of oxygen vacancies, has to be involved. In any case, the substantial changes in luminescent properties of the studied material can be expected. The electronic transitions of Tm^3+^ and Tb^3+^ ions are shown in Figure 2.

## 2. Materials and Methods 

### 2.1. Sample Preparation

#### 2.1.1. Powder Form

The LiMgPO_4_ powders of different dopants concentration were synthesized by a solid-state reaction in air. As the substrates, the lithium hydroxide (LiOH), hexahydrate magnesium nitrate (Mg(NO_3_)_2_·6H_2_O), and ammonium dihydrogen phosphate (NH_4_H_2_PO_4_) have been utilized. The synthesis of LMP compound occurs as a result of the following reaction:

(1) $$2\mathrm{LiOH}+2\mathrm{Mg}{\left({\mathrm{NO}}_{3}\right)}_{2}\cdot 6H_{2}O+2{\mathrm{NH}}_{4}H_{2}{\mathrm{PO}}_{4}\overset{\Delta T}{\to }2{\mathrm{LiMgPO}}_{4}+2{\mathrm{NH}}_{3}+4{\mathrm{NO}}_{2}+16H_{2}O+O_{2}$$

This chemical reaction was followed by several annealing cycles at the temperatures ranging from 200 to 750 °C. H_3_BO_3_ (boric acid) or Na_2_B_4_O_7_·10H_2_O (borax) was used for doping the phosphors with boron (B) ions. Tb_4_O_7_ and Tm_2_O_3_ oxides were used for doping the phosphors with terbium and thulium ions, respectively. The final product obtained in this process was next ground and sieved to achieve a grain size below 212 µm. 14 different compositions, regarding Tb^3+^ and Tm^3+^ dopants concentrations were of interest. The chemical composition of the studied LiMgPO_4_ crystal samples is given in Table 1.

#### 2.1.2. Crystal Form

The obtained LMP powders were next used to grow the crystals from the melt by the micro-pulling-down (MPD) method. This is a relatively new method of crystal growth which has been proposed for the first time in the mid-nineties of the XX century in Fukuda Laboratory, Sendai, Japan. In general, the main stage of the method is based on a pulling of the melted material in downward direction through the micro-capillary channel performed in the bottom of a specially designed conductive metal crucible [30,31]. For this purpose, the raw materials were loaded into the graphite crucible and melted inside of the inductive furnace. The Mo overlay was placed around the crucible in order to improve the heating conditions and thermal energy transfer to the raw material. The graphite after-heater and two layers of alumina ceramic thermal isolation were also applied in order to assure an appropriate temperature gradient within the growth zone. Finally, the melt was pulled down, using the iridium seed, at the constant rate of 0.2 mm/min with the presence of an inert gas atmosphere (Ar). The obtained rod-shaped crystals were around 3 mm in diameter and up to 60 mm in length. The as-grown crystals were cut into slices of around 3 mm in diameter and around 1 mm in thickness. The slices were weighted and all obtained results were recalculated regarding the mass of the samples.

### 2.2. XRD Phases Analysis

The X-ray diffraction patterns were measured for the studied powders and crystals using the Philips X’Pert pro diffractometer operating in a Bragg–Brentano geometry (XRD, Philips, Almeno, Netherlands). As the radiation source a Cu-anode X-ray lamp was used (monochromatic K_α1_ radiation of 1.5406 Å). Because a standard powder diffraction procedure was applied, the crystal samples had to be additionally powdered (with the agate mortar) before the measurements. The measurements were performed over 2-theta angle range from 15 to 75°. For collected data analysis the X’Pert HighScore Plus software have been utilized.

### 2.3. Irradiations and TL Measurements

Stimulated luminescence phenomena (such as e.g., thermoluminescence, TL), also commonly known as the main measurement techniques in radiation dosimetry, are presently increasingly used for material research. That is because of very high sensitivity of these techniques to defects in solids. The many possibilities in testing the properties of solids mean that the TL is nowadays considered as a research tool for investigations of luminescence mechanisms [32]. As the result of measurement, the so-called “glow-curve” is registered, which is a relationship between the temperature of the sample and the amount of luminescence light (expressed in arbitrary units) emitted by the sample at a given temperature. The peak-shape glow-curves are usually measured and each peak can be directly correlated to the trapping level in the structure of studied material. The exemplary glow-curves measured for the studied LMP compound can be seen further in the text (see in Section 3.2). For more detailed description of the TL technique please refer to [32,33].

Within this work, for TL measurements the automated Risø-TL/OSL-DA-20 reader was exploited. The applied detection system consisted of bialkali photomultiplier tube and a set of band pass filters chosen appropriately to cover the emission range of the studied samples. The DA-20 reader is also equipped with two sources of ionizing radiation, namely ^241^Am alpha particles and ^90^Sr/^90^Y beta radiation source. The detailed specification of the reader and its performance can be found in [34]. The measurements were performed from room temperature (RT) to 450 °C at the constant heating rate of 2 °C/s. During all measurements the volumetric flow rate of argon was around 0.2 l/min. All the measured glow-curves were next analyzed using a dedicated GlowVIEW software [35]. The TL response was investigated in terms of the amplitude of most prominent TL peak. The glow-curve measurements were performed with simultaneous registration of emission spectra, allowing to distinguish the spectral range of emission of the studied samples. For this purpose, the highly-sensitive Ocean Optics QE Pro spectrometer was connected to the Risø reader by a 400 µm core diameter optical fiber. The applied spectrometer allows to register the TL emission spectra over the wavelength range from 200 to 1000 nm with 4 nm resolution. The main advantage of the spectrometer over the applied commercial TLD reader is its flat spectral characteristic, so the measurements were performed without any additional optical filters. Therefore, the measured glow-curves are not affected by the spectral characteristics of the applied PMT tube and optical filters as is the case of the measurements performed with the Risø reader.

## 3. Results and Discussion

### 3.1. XRD Phases Analysis

**Figure 3:**
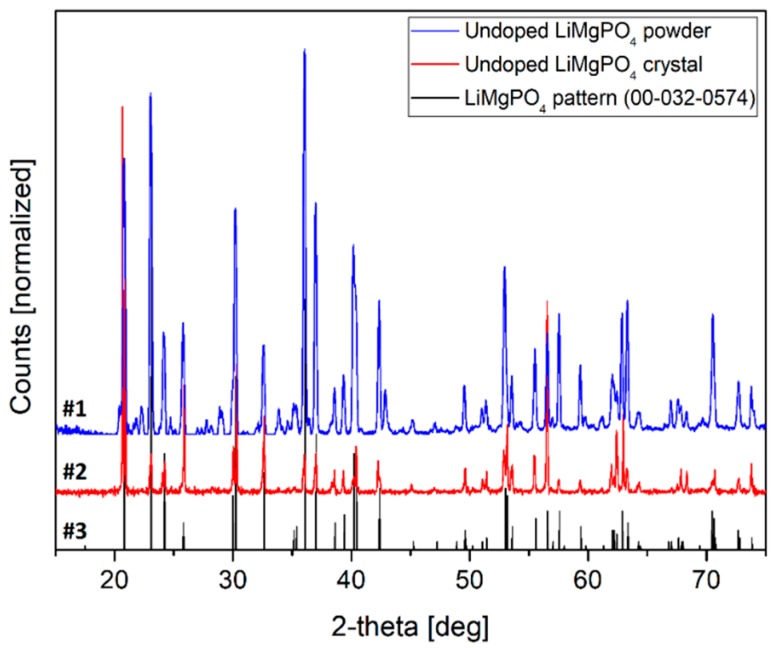
The XRD diffraction patterns of the undoped LiMgPO_4_ samples in form of powder (#1), crystal (#2) and reference pattern (#3).

The crystal structure of the final products, both synthesized powders and melt-grown crystals, was checked by the XRD measurements. Because a standard powder diffraction procedure was applied, the obtained crystal samples were additionally powdered before the measurements using the agate mortar. The exemplary XRD diffraction patterns (measured for the undoped LMP samples) are shown in Figure 3. The main criterion decisive on the presence of a given phase in the studied samples is the accordance of the Bragg reflections positions in the reference and the measured diffraction patterns. The analysis should also include a comparison of the intensities of the corresponding reflections, however, the decisive criterion is the accordance of the positions. The intensities should be comparable, however, some differences, resulting e.g., from the privilege arrangement of crystallites in the sample, are observed quite often. From Figure 3, it is clearly visible that the measured diffraction patterns remain in agreement with the reference pattern (Figure 3, curve #3), regarding the positions of the most of the reflections. Regarding the sensitivity differences, these may be additionally caused by different methods of samples preparation, namely solid-state synthesis (in case of powders) and high-temperature melt-growth (in case of crystals). The latter is in particular responsible for the introduction of structural defects like e.g., antisite defects (AD), which are most common in case of melt-growth techniques. In case of the crystal sample (Figure 3, curve #2), all Bragg peaks can be easily identified, what clearly indicates that no other phases are present in the sample.

**Figure 4:**
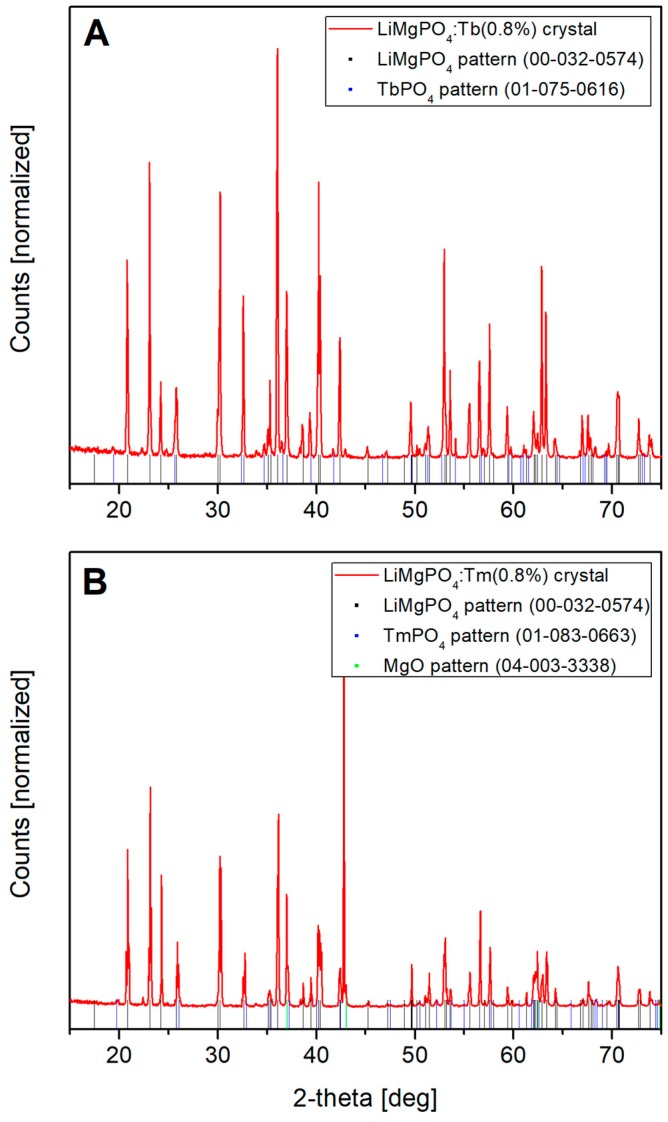
The XRD phase analysis performed for LMP:Tb (0.8 mol%, **A**) and LMP:Tm (0.8 mol%, **B**) crystals. Small amounts of TbPO_4_ and TmPO_4_ phases were found, respectively.

It should be noticed that similar results were obtained for other analyzed samples. Only in case of LMP:Tb (0.8 mol%) and LMP:Tm (0.8 mol%) small amounts of TbPO_4_ and TmPO_4_ phases were observed, respectively. Additionally, trace amounts of MgO phase were identified in the Tm-doped sample. These results are shown in Figure 4. The relative contribution of the terbium and thulium phosphate phases was evaluated by Rietveld method (using the Inorganic Crystal Structure Database (ICSD)) to 2 and 6 mass%, respectively. The other samples analyzed in this work were composed of clear LiMgPO_4_ phase.

### 3.2. TL Glow-Curve Shape Analysis

**Figure 5:**
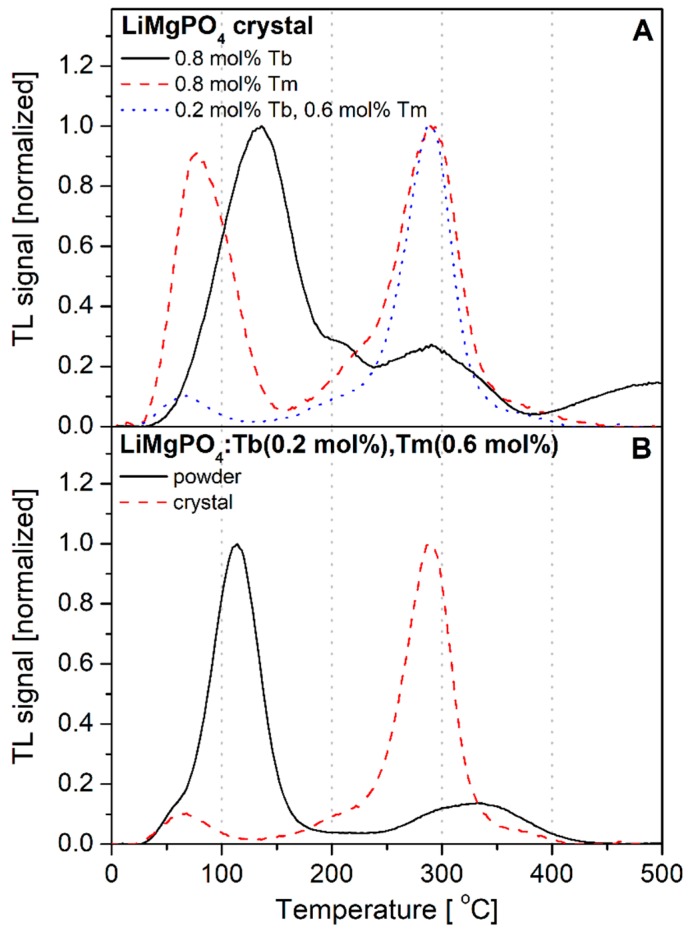
Panel (**A**): comparison of the glow-curves shape for the studied LiMgPO_4_ crystals doped with Tb (solid line), Tm (dashed line), and both Tb and Tm simultaneously (dotted line). Panel (**B**): comparison of the glow-curves shape for LiMgPO_4_:Tb(0.2),Tm(0.6) powder (solid line) and crystal (dashed line). All the glow-curves were measured after the samples’ irradiation with beta radiation.

It is important to realize that not all studied compounds are suitable for dosimetric applications. Some of them show more advantageous characteristics than the others and the decisive criterion can be e.g., the glow-curve shape. In Figure 5A, it is visible that the glow-curve measured for Tb-doped LMP crystal is dominated by low-temperature signal with a maximum below 150 °C (solid line). This may have a strong influence on fading properties for this material. Replacing Tb by Tm, one can highly increase the contribution of high-temperature signal (with maximum at around 290 °C), but the low-temperature part of the glow-curve remains significant (dashed line). However, applying both Tb and Tm as co-dopants of the opposite trapping nature, it is possible to suppress the low-temperature TL component by almost ten times (dotted line). It is also remarkable that the glow-curve shape for Tb, Tm co-doped material in crystal form is more advantageous as compared to the glow-curve measured for this same material in powder form. This can be seen in Figure 5B, which compares the TL glow-curves measured for the same material in powder (solid line) and crystal (dashed line) forms. The powders were obtained by solid-state synthesis according to a description given in Materials and Methods section and crystals were obtained by the MPD melt-growth technique. Figure 5B indicates that high-temperature crystallization (close to the melting point) significantly changes the distribution of TL-related structure defects within the studied material. The overall TL signal intensity is lower for crystals (not visible in Figure 5B, because of normalization), which could be considered a disadvantage; however, the very low contribution of low-temperature components suggests that the crystals are worthy of investigation.

**Figure 6:**
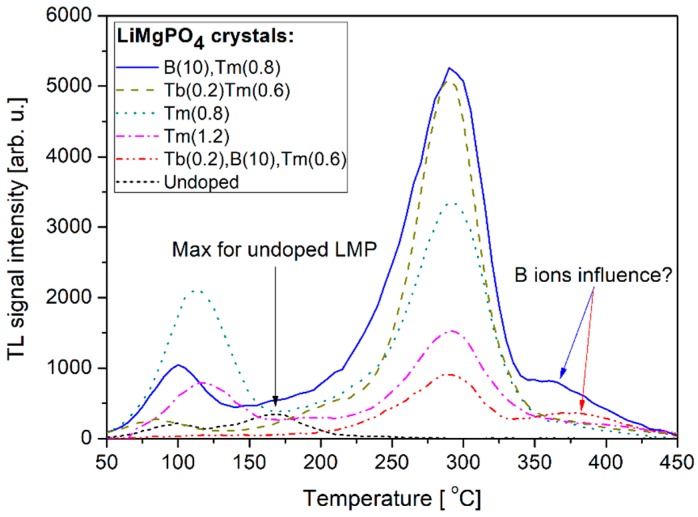
Comparison of the glow-curves measured for the studied LiMgPO_4_ crystals irradiated with beta radiation. The numbers in brackets denote the concentrations of dopants and are given in mol%.

Figure 6 compares TL glow-curves recorded for different concentrations of Tb, Tm, and B co-dopants. The curves were corrected regarding only the samples weights. It should be reminded here that the glow-curves measured for samples doped with a single Tb dopant were dominated by a low-temperature TL component, thus were excluded from further analysis even showing the highest signal intensity. Figure 6 allows for observation of some interesting features of the studied compounds. First of all, one can see that the undoped sample shows the lowest signal intensity with a maximum at around 160 °C. Introducing of Tm dopant causes additional defect-related energy levels to appear within the forbidden band-gap. These deeper energy levels are related to the shift of maximum thermoluminescence emission towards higher temperatures. Also, a strong luminescence enhancement is observed (about 15-times greater compared to the undoped sample) in the case of Tb(0.8), B(10) and Tb(0.2), Tm(0.6) samples. These two samples exhibited the highest signal intensity, practically at the same level, however, much a lower contribution of low-temperature signal was observed for Tb(0.2), Tm(0.6) doped crystal sample. The glow peaked at around 100–125 °C, which was probably connected to intrinsic defects energy levels, as it was visible for all analyzed samples, both RE-doped and undoped. Boron ions as co-dopants seem to have an influence on the high-temperature part of the glow-curve, above 350 °C, as an additional TL component was observed at this temperature range for boron co-doped samples. This, however, requires more detailed investigations which are out of the scope of this work.

### 3.3. TL Response Dependence on Dopant Concentration

**Figure 7:**
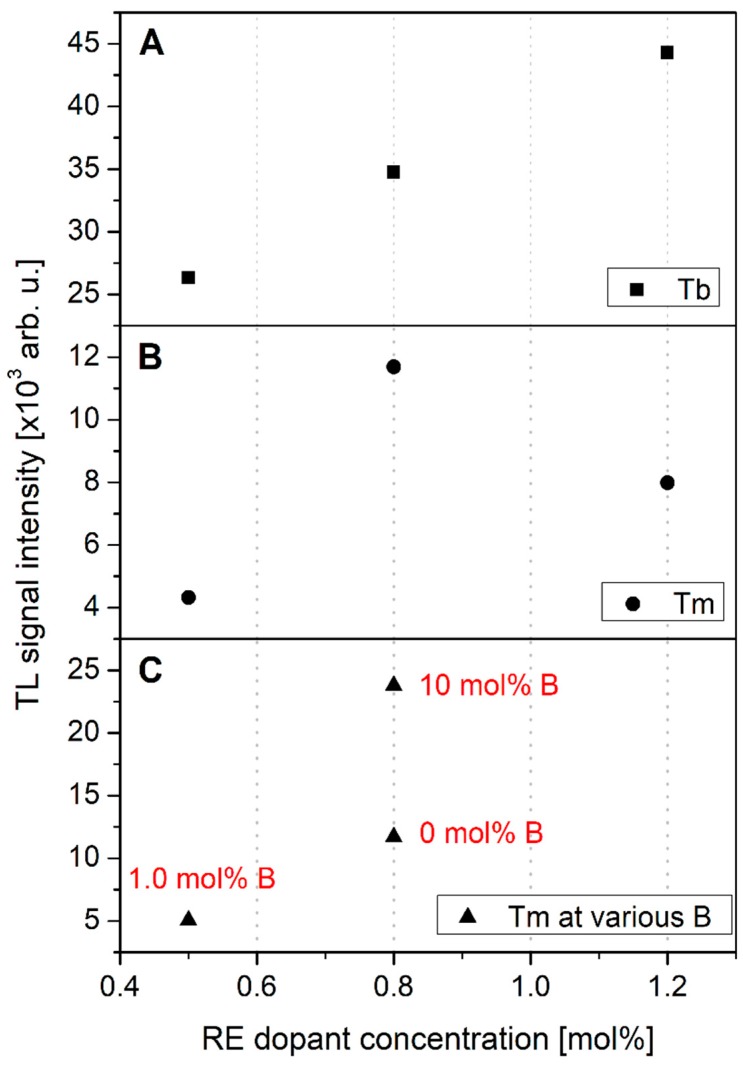
Thermoluminescence signal intensity as the function of the concentration of the RE doping ions: Tb (panel **A**), Tm (panel **B**), Tm at the presence of additional B co-doping ions (panel **C**). Note that in most cases the error bars are smaller than the size of measured data-points.

**Figure 8:**
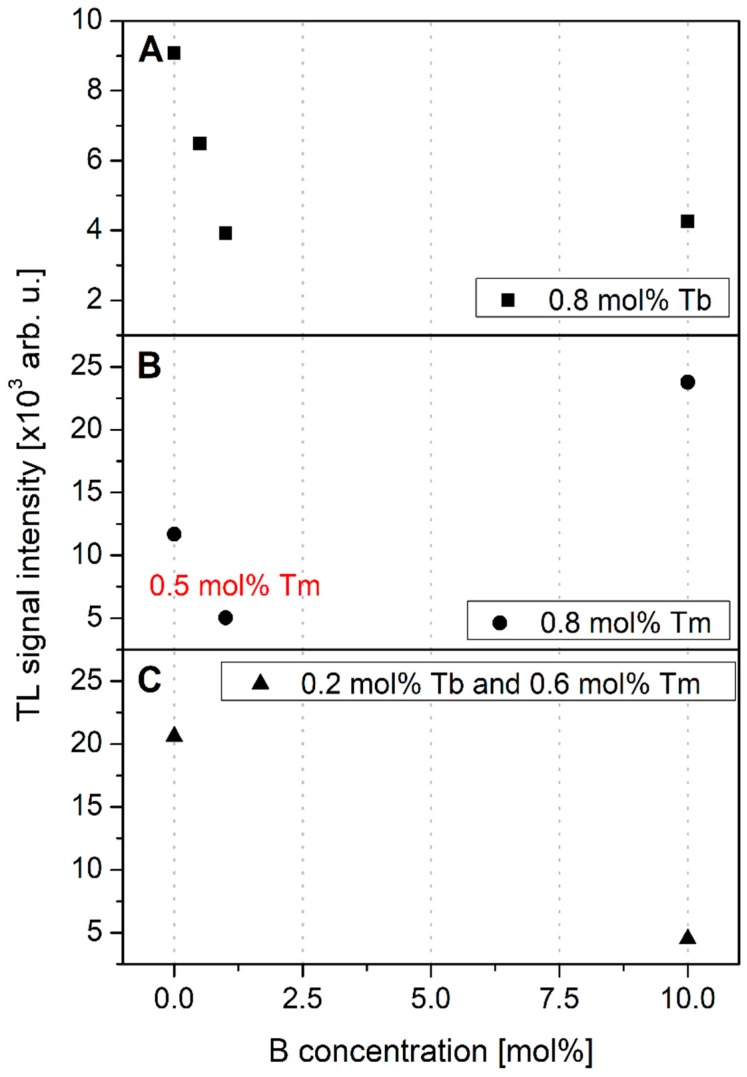
Thermoluminescence signal intensity as the function of B ions concentration in 0.8 mol% Tb-doped crystals (panel **A**), 0.8 mol% Tm-doped crystals (panel **B**) and 0.2 mol% Tb and 0.6 mol% Tm-doped crystals (panel **C**). Note that in most cases the error bars are smaller than the size of measured data-points.

As 14 different compositions have been investigated within this work, reliable dependences of TL response on RE dopant concentration were obtained. The obtained results were divided into following groups: (I) Single RE dopant concentration (Tb, Figure 7A; Tm, Figure 7B), (II) Tm dopant concentration at the presence of additional B co-doping ions (Figure 7C), (III) B dopant concentration at the presence 0.8 mol% of RE co-doping ions (Tb, Figure 8A; Tm, Figure 8B), and (IV) B dopant concentration at the presence of 0.8 mol% of Tb and 0.8 mol% of Tm ions (Figure 8C). It is important to note that in most cases the error bars are smaller than the size of measured data-points.

Figure 7 gives an important knowledge on TL responses of the studied compounds. First of all, over the studied range of Tb dopant concentration (panel A), the increasing trend of TL response was observed for increasing Tb content. The measured response increases in a quasi-linear way with no clearly visible maximum over the studied concentration range. This may suggest that higher Tb concentrations are also possible to synthesize and may be of interest for further investigations.

Somewhat different behavior was observed for Tm-doped crystals (panel B). In this case a very visible maximum was measured for 0.8 mol% Tm concentration. For higher Tm contents the intensity of luminescence strongly decreased. This may suggest the TL-related Tm trapping sites become saturated around this concentration and for higher Tm contents a concentration quenching phenomenon was observed. However, it was possible to increase the efficiency of 0.8 mol% Tm-doped samples by additional co-doping with B ions (panel C). The TL response intensity increased around two-fold when the B concentration increased to 10 mol%. These are, however, only two data-points, so it is not possible at this moment to obtain more profitable conclusions. More detailed analysis regarding different B concentrations in 0.8 mol% Tm-doped crystals is required to solve this issue.

Figure 8 shows thermoluminescence signal intensity as the function of B ions concentration in 0.8 mol% Tb-doped crystals (panel A), 0.8 mol% Tm-doped crystals (panel B), and 0.2 mol% Tb and 0.6 mol% Tm-doped crystals. Although a strong luminescence enhancement of Tm-doped crystals was observed by B ions co-doping, a completely opposite relationship was found for samples doped with Tb. This can be seen in Figure 8A. It is visible that luminescence signal intensity gradually decreased in a quasi-linear way as content of B ions increases up to 1 mol%. Further increasing the boron content did not cause significant changes in the intensity of the luminescence. Such a behavior may suggest that the introduction of B ions activated competitive recombination mechanisms responsible for luminescence quenching.

It is also remarkable that a similar destructive nature of B ions was measured for samples Tb, Tm co-doped. Figure 8C presents the results obtained for two groups of samples, both co-doped with Tb (0.2 mol%) and Tm (0.6 mol%), but to one of these groups 10 mol% of B ions was additionally introduced. One can see that luminescence intensity is around 4 times higher with no B-ion content. However, similar to the case in Figure 7C, more detailed analysis is required to obtain more convincing results.

It is also very interesting that LMP crystals doped with a single rare-earth dopant, like Tb or Tm, presented a completely opposite behavior after an additional co-doping with B ions (see Figure 8A,B). The possible explanation may be related to the opposite trapping nature of the studied RE dopants. According to an empirical model developed by Dorenbos [36,37,38] and further experimentally confirmed by Bos et al. [39,40,41] for a similar orthophosphate compound as studied in this work, divalent and trivalent lanthanides ground state energy levels locations, relative to the valence and conduction bands of the host, create a characteristic pattern (also called as a zigzag curve). Because the 4f electrons are shielded from host lattice by 5s and 5p orbitals, the zigzag pattern is almost independent of the host material. The ground state energy of Tb^3+^ relative to the top of the valence band allows for consideration of this dopant as a hole trapping and recombination center. The ground state energy location of Tm^2+^ ions is below the bottom of conduction band, thus Tm^3+^ ions may act as the electron traps [41]. A similar behavior was recently described for Tb^3+^ and Eu^3+^ (also considered as holes and electrons traps, respectively) ions doped mixed oxides crystals [42].

### 3.4. TL Emission Spectra Analysis

**Figure 9:**
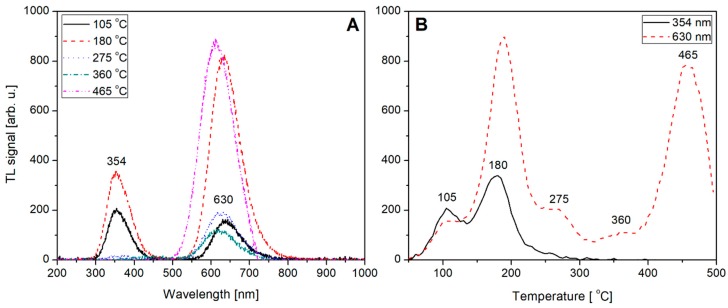
Thermoluminescence (TL) emission spectra (panel **A**) measured for the undoped LiMgPO_4_ crystal at different temperatures corresponding to the glow-peaks maxima. TL glow-curves (panel **B**) measured for the undoped LiMgPO_4_ crystal (irradiated with beta radiation) at different wavelengths corresponding to emission maxima.

**Figure 10:**
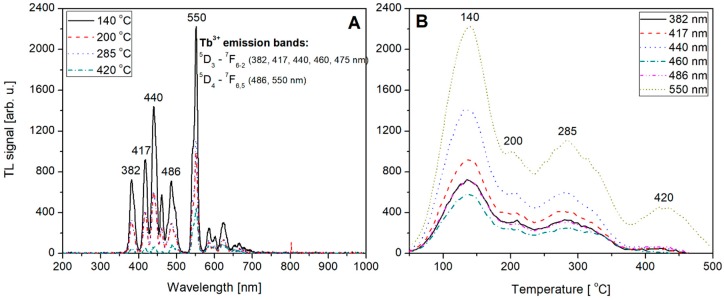
TL emission spectra (panel **A**) measured for Tb-doped (0.8) LiMgPO_4_ crystal at different temperatures corresponding to the glow-peaks maxima. TL glow-curves (panel **B**) measured for the same Tb-doped LiMgPO_4_ crystal (irradiated with beta radiation) at different wavelengths corresponding to emission maxima.

**Figure 11:**
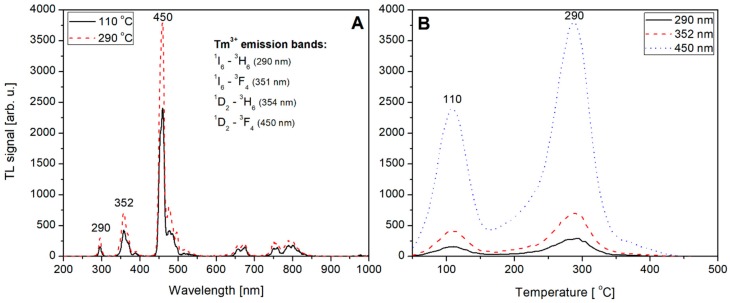
TL emission spectra (panel **A**) measured for Tm-doped (0.8) LiMgPO_4_ crystal at different temperatures corresponding to the glow-peaks maxima. TL glow-curves (panel **B**) measured for the same Tm-doped LiMgPO_4_ crystal (irradiated with beta radiation) at different wavelengths corresponding to emission maxima.

**Figure 12:**
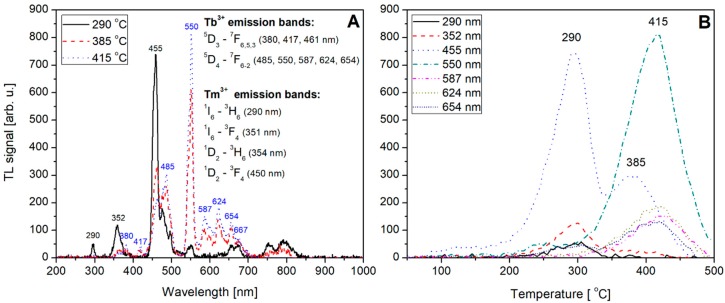
TL emission spectra (panel **A**) measured for Tb (0.2), Tm (0.6) double doped LiMgPO_4_ crystal at different temperatures corresponding to the glow-peaks maxima. TL glow-curves (panel **B**) measured for the same Tb (0.2), Tm (0.6) double doped LiMgPO_4_ crystal (irradiated with beta radiation) at different wavelengths corresponding to emission maxima.

Not-normalized TL emission spectra measured for the undoped, Tb (0.8) doped, Tm (0.8) doped and Tb, Tm (0.2, 0.6) doped LiMgPO_4_ crystals are shown on Figure 9A, Figure 10A, Figure 11A, and Figure 12A, respectively. The spectra were measured at different temperatures corresponding to the glow-curves maxima. Corresponding glow-curves measured at different wavelengths (corresponding to characteristic emission maxima) are shown on Figure 9B, Figure 10B, Figure 11B, and Figure 12B, respectively. It should be mentioned here that the glow-curves were measured with a spectrometer, not with photomultiplier tube, to make the glow-curves independent of the spectral characteristics of all the applied elements of spectral path (such as optical filters and PMT). In other words, there were no other optical elements placed between the sample and spectrometer, thus the measured spectra are only influenced by internal characteristic of the applied spectrometer.

Figure 9A,B show an intrinsic defect related luminescence measured for the undoped LiMgPO_4_ crystal. Two broad bands located at around 354 and 630 nm are clearly visible. This red-range emission component seems especially interesting, as its intensity is practically twice as high as the close UV-range emission component. At a low-temperature range, up to 105–110 °C, both emission bands present comparable intensity. For higher temperatures, the red-range emission band increases rapidly and reaches its first maximum at around 180 °C. With a further increase in temperature the intensity of this emission band decreases, showing two less-intensive local maxima at around 275 and 360 °C, and then starts increasing again, reaching its second maximum at 465 °C. The intensity of this second maximum is practically at the same level as the maximum at 180 °C. Nevertheless, this red-range luminescence can be observed only by using spectrometer or red-sensitive PMT. Using a standard Risø TL/OSL reader equipped with EMI 9235QB PMT, this luminescence cannot be investigated. Fortunately, the close UV-range emission band fits very well to the reader characteristic and can be efficiently measured with a Hoya U340 optical filter.

Figure 10A shows the emission spectra measured for Tb-doped (0.8 mol%) LMP crystal. In this case the intrinsic defects related luminescence is not observed and all visible peaks can be easy ascribed to the well-known pattern of Tb^3+^ emission. These are in particular the ^5^D_3_ → ^7^F_6-2_ transitions corresponding to 382, 417, 440, 460, and 475 nm, respectively, and also ^5^D_4_ → ^7^F_6,5_ transitions corresponding to 486 and 550 nm, respectively. It should be also noted that the less-intensive ^5^D_4_ → ^7^F_4-1_ transitions corresponding to 587, 624, 654, and 667 nm are also visible. The obtained pattern clearly indicates that the emission is from Tb^3+^ hole trapping dopant. The corresponding glow-curves at emission maxima are shown on Figure 10B. Over a whole range of wavelengths only intensity of the glow-curve changes reaching the maximum for 550 nm.

Similar observations can be made for Tm-doped (0.8 mol%) LMP crystal. The emission spectra in this case are shown in Figure 11A showing a well-known pattern of Tm^3+^ emission. These are in particular the ^1^I_6_ → ^3^H_6_ and ^1^I_6_ → ^3^F_4_ transitions which correspond to 290 and 351 nm, respectively. The most intensive emission peak located at 450 nm corresponds to ^1^D_2_ → ^3^F_4_ transition. It should be mentioned here for clarification that emission peak at 352 nm in Figure 11A results probably from the superposition of ^1^I_6_ → ^3^F_4_ (351 nm) and ^1^D_2_ → ^3^H_6_ (354 nm) transitions which cannot be clearly separated because of too low spectrometer resolution (4 nm). The measured spectra indicate that the emission is from Tm^3+^, which suggests that recombination of charge carriers took place at Tm-related trapping center. The corresponding glow-curves at emission maxima are shown on Figure 11B. Over a whole range of wavelengths only the intensity of the glow-curve changes reaching a maximum at 450 nm.

An interesting situation was found for LMP crystals double doped by Tb and Tm presenting an opposite trapping nature. The spectra presented in Figure 12A show the emission peaks which are characteristic for both Tb and Tm co-dopants and were described in detail in Figure 10A and Figure 11A. The obtained results are in good agreement with findings of Bos et al. [37] for a similar orthophosphate compound. This may confirm the hypothesis that charge carriers occupying the Tb and Tm trapping sites of similar depths may become mobile at the same temperature range giving rise to both Tb^3+^ and Tm^3+^ emission. This was postulated by Bos et al. for YPO_4_:Tb,Tm compound [37]. For LiMgPO_4_ crystals double doped with Tb and Tm, similar phenomena have been confirmed by the results of this work. Moreover, an additional temperature dependence of Tb^3+^ and Tm^3+^ emission was evaluated. As can be seen in Figure 12A, at a temperature range up to around 290 °C most of the emission is from Tm^3+^ (black solid line). The most intensive peak at 455 nm seems to be a superposition of ^1^D_2_ → ^3^F_4_ transition of Tm^3+^ (450 nm) and ^5^D_3_ → ^7^F_3_ transition of Tb^3+^ (460 nm), however the emission of Tm^3+^ is dominant. The most intensive ^5^D_4_ → ^7^F_5_ transition of Tb^3+^ (550 nm) is very weak-visible. At further increase in temperature, the emission from Tm^3+^ decreases with a simultaneous increase of Tb^3+^ emission. This was clearly shown by red-dashed and blue-dotted lines in Figure 12A, where characteristic Tm^3+^ transitions at 290, 351, and 354 nm are completely not visible, 450 nm transition is strongly reduced and characteristic Tb^3+^ emission with maximum at 550 nm became dominant. The corresponding glow-curves at the wavelengths of emission maxima are shown in Figure 12B. The glow-curve at 455 nm consists of two broad peaks at 290 and 385 °C. The first one is related to Tm^3+^ and the second one to Tb^3+^ emission. The glow-curve at 550 nm is related mostly to Tb^3+^ emission, which is however inconsistent with the glow-curve measured for LMP crystals doped with a single Tb dopant (see Figure 10B). This probably results from the presence of co-doping Tm^3+^ ions of an opposite trapping nature.

The obtained results tend to confirm the thesis on mobility of charge carriers released from Tb hole trapping sites and Tm electron trapping sites at similar temperature range giving rise to the characteristic emission of both dopant types. However, the observed temperature dependence suggests that in case of LiMgPO_4_ compound the situation looks somewhat different from this in YPO_4_. First of all, it seems that in case of LiMgPO_4_ host the Tm-related electron trap depth is greater than the Tb-related hole trap depth. That is why mainly Tm^3+^ emission is observed at a low-temperature range. At a higher temperature, the electrons at Tm^3+^ sites start to be released giving rise to the emission from Tb-related recombination centers. The emission from Tm^3+^ centers decreases simultaneously. It seems that at the highest temperatures, emission takes place from Tb^3+^ recombination centers, but only from deeper ^5^D_4_ level related traps which have not been emptied at lower temperatures. Confirmation of this thesis needs further investigations and a separate paper will be devoted to this issue.

## 4. Concluding Remarks

The influence of Tb^3+^ and Tm^3+^ trivalent rare-earth ions co-doping on luminescent properties enhancement of LiMgPO_4_ crystal host was investigated in this work. Fourteen different compositions, regarding Tb^3+^ and Tm^3+^ dopants concentrations, were of interest. Luminescent properties of the obtained crystals were studied by thermoluminescence method. The XRD measurements were performed for all studied samples confirming very good phase purity. Only in two cases the trace amounts of the phase other than LiMgPO_4_ were detected. The most favorable properties and also the highest luminescence enhancement (15 times higher as compared to the undoped sample) were measured for Tb and Tm double doped crystals. A similar luminescence level can be also obtained for Tm, B co-doped samples. However, in this case a low-temperature TL component has a significant contribution. The measured luminescent spectra showed a typical emission of Tb^3+^ and Tm^3+^ ions of an opposite trapping nature, namely the holes and electrons trapping sites, respectively. The most prominent transitions of ^5^D_4_ → ^7^F_3_ (550 nm for Tb^3+^) and ^1^D_2_ → ^3^F_4_ (450 nm for Tm^3+^) were observed. The opposite trapping nature of the studied RE dopants was strongly manifested in the measured glow-curves and emission spectra. It was also found that Tb^3+^ and Tm^3+^ emissions show temperature dependence in case of double doped LiMgPO_4_ crystal, which was not observed for any other sample. This temperature dependence causes that at low temperature range (up to about 290 °C) Tm^3+^ emission is dominant. At higher temperatures, the electrons occupying Tm^3+^ sites start to be released giving rise to the emission from Tb-related recombination centers. Simultaneously, the emission from Tm^3+^ related centers decreases. It seems that at the highest temperatures (above 400 °C) emission takes place from Tb^3+^ recombination centers, but only from deeper ^5^D_4_ level related traps which have not been emptied at lower temperatures. Confirmation of this thesis needs further investigations and a separate paper will be devoted to this issue.

## Figures and Tables

**Table 1:** Chemical composition of the samples studied in this work.

Host	Dopants Concentration [mol%]			Phase Purity [mass%] *	
	Tb	Tm	B	LiMgPO_4_	Other Phases
LiMgPO_4_	-	-	-	100	-
	0.5	-	-	100	-
	0.8	-	-	98	2 (TbPO_4_)
	1.2	-	-	100	-
	-	0.5	-	100	-
	-	0.8	-	94	6 (TmPO_4_)
	-	1.2	-	100	-
	0.8	0.5	-	100	-
	0.8	1.0	-	100	-
	0.8	10	-	100	-
	-	0.5	1.0	100	-
	-	0.8	10	100	-
	0.2	0.6	-	100	-
	0.2	0.6	10	100	-

* checked within this work by the XRD measurements.
